# Role of micronutrition in patients with oral cancer and nutritional intervention strategies

**DOI:** 10.3389/fnut.2025.1616344

**Published:** 2025-07-24

**Authors:** Yunwei Fan, Yuling Feng, Wenxin Liu

**Affiliations:** ^1^School of Medicine, Jiangsu University, Zhenjiang, Jiangsu, China; ^2^Department of ICU, Xinghua People's Hospital Affiliated to Yangzhou University, Xinghua, Jiangsu, China; ^3^Center for Intravenous Infusion Therapy and Nursing, Affiliated People's Hospital of Jiangsu University, Zhenjiang, Jiangsu, China; ^4^Department of Internal Medicine, Xinghua People's Hospital Affiliated to Yangzhou University, Xinghua, Jiangsu, China

**Keywords:** oral cavity cancer, micronutrients, inflammation, immunity, nutritional intervention, oxidative stress

## Abstract

Oral cavity cancer exhibits high mortality rates with conventional therapies often causing nutritional complications. Emerging evidence highlights the critical role of micronutrients in modulating oxidative stress, a key driver of carcinogenesis in precancerous lesions including oral lichen planus, leukoplakia and submucous fibrosis. Zinc deficiency impairs antioxidant defenses while copper excess promotes angiogenesis. Selenium maintains redox balance through selenoproteins and vitamins A, E and C exhibit chemopreventive effects through reactive oxygen species scavenging and immunomodulation. Immunonutrition strategies incorporating omega-3 fatty acids and arginine demonstrate benefits in postoperative outcomes. This review summarizes the mechanistic roles of antioxidant micronutrients including zinc, copper, selenium and vitamins A, D, E, C and B complex in oral squamous cell carcinoma pathogenesis and explores personalized nutritional interventions to enhance treatment tolerance and quality of life. Optimizing micronutrient status represents a promising adjuvant approach in comprehensive oral cancer management.

## 1 Introduction

Oral cavity cancer is associated with significant mortality, with fatality rates approaching 50% of diagnosed cases (1, 2). Surgery, radiotherapy, and chemotherapy frequently induce adverse effects such as dysphagia, taste alterations, and oral mucositis (3–5). These treatment-related complications, if not properly managed, can lead to nutritional deterioration, potentially establishing a detrimental cycle that may compromise clinical outcomes (6). Evidence suggests that appropriate nutritional interventions may provide multiple benefits, including enhancing the treatment tolerance, reducing treatment interruptions, improving therapeutic efficacy, supporting disease control, and facilitating post-treatment recovery (7).

In recent years, with the continuous advancement of micronutrient research techniques, an increasing body of evidence suggests a link between the occurrence of oral cancer and antioxidant micronutrients (8). Oxidative stress refers to a state in which the balance between the formation of reactive oxygen species (ROS) and antioxidant defenses is disrupted, and it plays a role in the development of precancerous lesions such as oral lichen planus (OLP), oral leukoplakia (OLK), and oral submucous fibrosis (OSF) (9–12). The body’s defense against oxidative stress can be achieved through the antioxidant activity of micronutrients (minerals and vitamins) and the interconnected systems of enzymes, which can eliminate or inhibit the formation of free radicals or repair damage caused by free radicals, thereby protecting the body from the harmful effects of oxidative stress and potentially preventing disease onset (13, 14). Therefore, nutritional management, as part of the comprehensive treatment plan for oral cancer, holds significant importance in improving patient prognosis and enhancing quality of life (15). This review explores the relationship between the antioxidant mechanisms of micronutrients and the development of oral cavity cancer, as well as their potential therapeutic applications.

## 2 Antioxidant role of minerals in oral cancer development

### 2.1 Zinc

Zinc, an essential trace element, is widely distributed throughout oral ecosystems, including dental plaque, saliva, dental structures, and mucosal tissues, where it serves as a critical biological reservoir (16). This micronutrient plays a pivotal role in multiple physiological processes, including cellular proliferation, immune regulation, collagen synthesis, and tissue repair (17). Notably, clinical studies have established a significant association between zinc deficiency and increased susceptibility to oral cancer (18), with tumor patients frequently exhibiting depleted zinc levels, likely due to its consumption during fibrotic processes, inflammatory responses, and free radical-scavenging activities (19). The therapeutic and prophylactic effects of zinc are mediated through several distinct mechanisms: First, as a cofactor for copper-zinc superoxide dismutase (SOD), zinc exhibits anticancer activity, particularly in OSF (20). Second, it modulates collagen metabolism by suppressing lysyl oxidase (LOX)-mediated collagen deposition while simultaneously promoting matrix metalloproteinase (MMP)-dependent degradation, thereby enhancing mucosal flexibility and alleviating clinical symptoms (21–24). Third, by upregulating glutathione levels, zinc synergizes with vitamins A and C to maintain epithelial integrity and reduce mucosal discomfort (25, 26). Furthermore, emerging evidence suggests that zinc reinforces mucosal barrier function through immunomodulatory effects and oxidative stress mitigation (27, 28). Research indicates that zinc supplementation physiologically modulates immune responses by suppressing excessive activation, while its depletion during severe infections leads to widespread upregulation of NF-κB signaling. *In vitro* experiments reveal that zinc downregulates NF-κB-mediated pathways along with associated pro-inflammatory cytokines, including TNF-*α* and IL-1β. Concurrently, it enhances transcriptional activity of A20 and PPAR-α, both of which are zinc-dependent regulators exhibiting anti-inflammatory functions (27).

In the pathogenesis of OLP, a condition marked by cytotoxic T-cell activation, MMP dysregulation, cyclooxygenase-2 (COX-2) overexpression, and redox imbalance, zinc demonstrates significant protective effects (29). As MMPs depend on zinc for their activation, zinc exerts precise control over inflammatory responses by inhibiting MMP-1-mediated lymphocyte infiltration and preventing MMP-9-induced basement membrane disruption (22, 23). Additionally, zinc functions as a potent reactive oxygen species (ROS) scavenger, suppressing COX-2/prostaglandin E2 (PGE2) signaling and thereby attenuating oxidative stress-driven inflammation in OLP patients (30). Collectively, zinc exerts its anti-carcinogenic and anti-inflammatory effects through multifaceted mechanisms, including modulation of the SOD/MMP axis, inhibition of COX-2–PGE2 signaling, and maintenance of redox homeostasis and epithelial integrity. These mechanistic pathways are strongly correlated with enhanced mucosal healing and clinical symptom relief in patients with oral cancer and OLP.

### 2.2 Copper (cu)

Copper ions serve as a critical micronutrient in numerous oxidoreductases including cytochrome oxidase and tyrosinase, essential for cellular homeostasis and biological functions (31, 32). While physiologically important, dysregulated copper metabolism exhibits dual roles in oral pathogenesis. On one hand, copper deficiency impairs SOD1 activity, compromising antioxidant defenses and leading to oxidative stress accumulation (33). On the other hand, elevated copper levels in biological fluids have been consistently associated with premalignant conditions and squamous cell carcinomas, particularly in OSF, a condition with high malignant transformation potential (34, 35). The oncogenic properties of copper involve multiple interconnected mechanisms: (1) activation of LOX-mediated collagen deposition, driving OSF progression (36); (2) induction of ROS-dependent growth genes (c-fos, c-jun); (3) stimulation of angiogenic factors (VEGF, b-FGF); and (4) promotion of proliferative signaling pathways (37–39). Notably, the copper-CER-SOD1 axis forms a critical regulatory network, where ceruloplasmin facilitates copper delivery for SOD1 biosynthesis, while SOD1 upregulation conversely depletes circulating copper pools (20). This delicate balance is further evidenced by studies showing that copper deficiency reduces antioxidant enzyme activities (GSH-Px, SOD1) while increasing oxidative markers (LPO, MDA), effects reversible upon copper supplementation (40, 41). The therapeutic potential of copper modulation is underscored by preclinical studies demonstrating that chelation therapy can simultaneously target multiple oncogenic pathways, including ROS reduction, SOD1 inhibition, and suppression of angiogenic factors (VEGF, MMP-9) (41, 42). However, current evidence remains limited to xenograft models, highlighting the need for clinical investigations in oral cavity cancers (43, 44). Importantly, dose–response studies reveal a narrow therapeutic window, with micromolar copper iron concentrations stimulating keratinocyte proliferation by ROS accumulation (45), emphasizing the necessity to precisely define physiological ranges that maintain redox homeostasis without inducing either deficiency or toxicity.

### 2.3 Selenium (se)

Selenium (Se), an essential trace element, functions through selenoproteins including glutathione peroxidase (GSH-Px) and thioredoxin reductase (TxRs), which serve as pivotal antioxidants (46). These proteins exhibit organelle-specific localization and tissue-dependent expression patterns, with activity sensitive to Se availability (47). Epidemiological evidence associates Se deficiency with elevated cancer risk due to impaired selenoprotein function (48). In lichen planus (LP), serum Se levels inversely correlate with lesion severity and chronicity (49). Notably, OLP demonstrates reduced Se levels in malignant progression, suggesting its chemopreventive role (50). Mechanistically, Se insufficiency diminishes GPX-1 activity, compromising H₂O₂ detoxification post-SOD2 reaction, thereby accelerating neoplastic transformation in OLP (51). The selenium-GPX axis restores H₂O₂ detoxification and suppresses pro-inflammatory cytokines via inhibition of NF-κB transcriptional activity (52). These pathways correlate with reduced OLP recurrence, improved epithelial repair, and reduced mucosal pain, demonstrating both molecular and clinical relevance. Beyond antioxidant effects, Se modulates immune responses and oxidative stress (53, 54). At the molecular level, Se suppresses NF-κB binding to cytokine gene promoters to mitigate inflammation, including TNF-*α*, IL-1, and IL-6 (55, 56). It also normalizes CD3^+^/CD4^+^ and CD4^+^/CD8^+^ ratios and Th1/Th2 balance, reducing OLP recurrence (57, 58). OLP pathogenesis involves ROS amplification, where ROS overproduction by CD4^+^ T cells perpetuates keratinocyte lipid membrane damage and localized inflammation. Se interrupts this cycle by neutralizing H₂O₂ and organic peroxides, preserving mucosal integrity (55).

### 2.4 Vitamin A and vitamin E

Vitamin A encompasses fat-soluble compounds, including retinol, retinoic acid, retinal, and carotenoids, that play crucial roles in modulating epithelial keratinization, inflammatory responses, and immune function (59–61). As a key antioxidant within the glutathione peroxidase system, vitamin E (*α*-tocopherol) effectively mitigates oxidative membrane damage by neutralizing ROS (62). While both vitamins exhibit lipid peroxidation inhibitory effects, emerging evidence suggests potential antagonistic interactions when administered concurrently (63). Clinical studies have established a strong correlation between deficiencies in these antioxidants and an elevated risk of oral cavity carcinogenesis, particularly in OLK and OLP progression (64–66). The pathogenesis involves tobacco-and betel nut-derived carcinogens that generate excessive ROS and malondialdehyde (MDA), leading to cytotoxic and genotoxic effects that promote mucosal malignant transformation (67). Antioxidant supplementation demonstrates therapeutic potential, with carotenoids showing particular efficacy in precancerous lesion regression (68). Notably, vitamin A or *β*-carotene supplementation reduces OLK lesion size and nuclear abnormalities, even with persistent carcinogen exposure (69). For refractory OLP cases, isotretinoin (9-cis retinoic acid) exhibits clinical efficacy, potentially through retinoic acid receptor activation or AP-1 pathway suppression, though the mechanisms underlying treatment resistance require further investigation (70, 71). *α*-Tocopherol demonstrates significant regulatory effects on free radicals and lipid peroxides in precancerous conditions (72). A recent network meta-analysis identified lycopene combined with vitamin E as the most effective intervention for OSF (73). However, the clinical application of systemic vitamin A therapy remains limited by its transient efficacy and notable adverse effects, including cheilitis, mucosal pigmentation, and impaired wound healing (74). These limitations underscore the need for developing safer vitamin A derivatives to enable sustained chemoprevention strategies in oral potentially malignant disorders.

### 2.5 Vitamin C

Vitamin C (L-ascorbic acid) is a potent water-soluble antioxidant that plays a crucial role in neutralizing organic free radicals and protecting biological membranes from oxidative damage (75). Its antioxidant mechanism involves two key aspects: direct radical scavenging and synergistic interaction with other antioxidants. Notably, vitamin C regenerates *α*-tocopherol from oxidized vitamin E, thereby restoring the antioxidant capacity of vitamin E (76, 77). In the context of carcinogenesis, where reactive oxygen/nitrogen species (ROS/RNS) induce significant DNA damage, vitamin C demonstrates diagnostic potential when combined with other biomarkers. Studies show that the combination of vitamins C/E significantly improves diagnostic sensitivity for oral precancerous lesions compared to using single biomarkers alone (78). The unique solubility properties of vitamin C enable its antioxidant action in both intracellular and extracellular compartments, effectively mitigating oxidative stress induced by infections (79). Furthermore, vitamin C exhibits a bimodal activity pattern through dose-dependent modulation of redox-sensitive signaling pathways, including NF-κB and MAPK cascades. These molecular interactions can lead to either DNA repair activation or cytotoxic effects, depending on the concentration of vitamin C, highlighting its complex role in cellular redox regulation. Nicolae et al. observed reduced urinary vitamin C in infected lichen planus (LP) patients, correlating with disease severity (79). Animal studies reveal elevated ascorbate in immune cells, bolstering infection resistance (79). Abdolsamadi et al. reported higher salivary MDA and lower antioxidants, such as vitamins A/E, in erosive OLP patients, linking OS to lesion susceptibility (63). Vitamin C also modulates OS-driven metabolic pathways. Depletion elevates ROS, oxidizing DNA (8-hydroxydeoxyguanosine), proteins (carbonyls), and lipids (8-iso-PGF2α), while altering glucose/cholesterol metabolism, enhancing cancer invasiveness (80). Importantly, Vitamin C exhibits concentration-dependent “bimodal” behavior. At physiological concentrations, it functions as an antioxidant, quenching ROS and stabilizing cell membranes (81). However, at pharmacologic or supraphysiological doses, it reduces transition metal ions such as Fe^3+^ to Fe^2+^ or Cu^2+^ to Cu^+^, facilitating Fenton-like reactions that produce hydrogen peroxide (H₂O₂) and hydroxyl radicals *in situ* (82, 83). This pro-oxidant effect selectively induces oxidative stress in cancer cells, which often have impaired catalase activity and a weakened antioxidant defense system, leading to DNA strand breaks, mitochondrial dysfunction, and apoptosis. This mechanism underpins the cytotoxic activity of high-dose Vitamin C in tumor settings (80).

### 2.6 Antioxidant effects of other vitamins

#### 2.6.1 Vitamin B complex

The Vitamin B complex consists of eight water-soluble vitamins: thiamine (VB1), riboflavin (VB2), niacin (VB3), pantothenic acid (VB5), pyridoxine (VB6), biotin (VB7), folate (VB9), and cobalamin (VB12). These vitamins are interconnected in their roles in protein, lipid, and nucleic acid synthesis, metabolism, and immune defense (84). Each B vitamin has demonstrated considerable antioxidant activity (85). Chen et al. (85) observed a significant association between anemia due to hemoglobin, iron, or vitamin B12 deficiencies and elevated homocysteine levels, with an increased prevalence of erosive OLP. Vitamin B12 and iron deficiencies, which lead to anemia, reduce the oxygen supply to the oral mucosal tissues, causing atrophy. Elevated homocysteine levels in erosive OLP patients contribute to OS, promoting thrombosis in small arteries supplying the oral epithelium, thereby compromising the epithelial barrier and increasing the frequency of OLP lesions. Studies indicate that elevated homocysteine levels in OLP patients correlate with deficiencies in vitamin B6, B12, and folate, and this increase has become a key marker of the Vitamin B complex’s involvement in antioxidant stress responses (86). Although empirical supplementation of B vitamins has alleviated subjective symptoms in some cases, studies show that deficiencies in B1, B6, C, folate, and carotenoids are not primary contributors to OLP pathogenesis. Furthermore, no complete recovery was observed in any patients after two months of intensive B vitamin supplementation (87).

#### 2.6.2 Vitamin D

Vitamin D is a fat-soluble vitamin that, through its metabolites such as 7-dehydrocholesterol, calcidiol, vitamin D2, and calcitriol, exhibits antioxidant properties by reducing lipid peroxidation (88). Existing research has established a close relationship between vitamin D deficiency and an increased risk of OLP (89). The active form of vitamin D, 1,25-dihydroxyvitamin D_3_ (1,25(OH)_2_D_3_), exerts its biological effects primarily through the vitamin D receptor (VDR), a nuclear receptor expressed in various epithelial cells (89). *In vitro* studies using HaCat cell models demonstrated that 1,25(OH)_2_D_3_, via VDR, can attenuate lipopolysaccharide-induced inflammatory cytokine expression by modulating the NF-κB signaling pathway, thereby reducing inflammation associated with OLP (90). Additionally, vitamin D plays a crucial role in mitigating DNA oxidative damage in mucosal tissues. Supplementation with exogenous vitamin D has been shown to significantly improve oxidative stress markers in patients with ulcerative colitis, including oxidized low-density lipoproteins, lipid peroxides, MDA, and superoxide dismutase, contributing to the repair of intestinal mucosal oxidative damage. Given that the oral cavity is part of the digestive tract and expresses VDR in keratinocytes, the binding of vitamin D to VDR in these cells can reduce oxidative stress levels and clear ROS to facilitate the repair of damaged oral mucosal barriers and promoting lesion healing (91). The 25(OH)_2_D_3_-VDR signaling pathway plays a protective role in maintaining the integrity of oral mucosal tissues, suggesting that vitamin D supplementation may serve as a potential strategy for managing OLP lesions (Table 1).

**Table 1:** Key antioxidant micronutrients in oral cancer pathogenesis and prevention.

Micronutrient	Biological functions	Association with oral cancer	Mechanistic roles	Therapeutic potential
Zinc	Cellular proliferation, immune modulation, collagen synthesis	Deficiency linked to higher cancer risk	SOD cofactor (antioxidant); LOX inhibition (anti-fibrotic); Glutathione elevation	Improves mucosal integrity, reduces OLP inflammation
Copper	Oxidoreductase cofactor, angiogenesis regulation	Elevated in premalignant lesions	ROS generation; LOX activation (pro-fibrotic); Angiogenic factor induction	Chelation therapy shows promise in xenografts
Selenium	Selenoprotein synthesis (GSH-Px, TxRs)	Deficiency correlates with malignant progression	H₂O₂ detoxification; NF-κB suppression; Th1/Th2 balance regulation.	Superior to steroids for long-term OLP management
Vitamin A	Epithelial differentiation, immune function	Deficiency increases OPMD risk	Retinoic acid receptor activation; AP-1 suppression.	Limited by toxicity; derivatives needed
Vitamin E	Lipid peroxidation prevention	Combined with lycopene most effective for OSF	Free radical scavenging; Membrane protection.	Synergistic with other antioxidants
Vitamin C	Water-soluble radical scavenger	Low levels in erosive OLP	Vitamin E regeneration; DNA damage prevention	Bimodal activity (pro/antioxidant)

## 3 Nutritional intervention strategies for oral cancer patients

**Figure 1:**
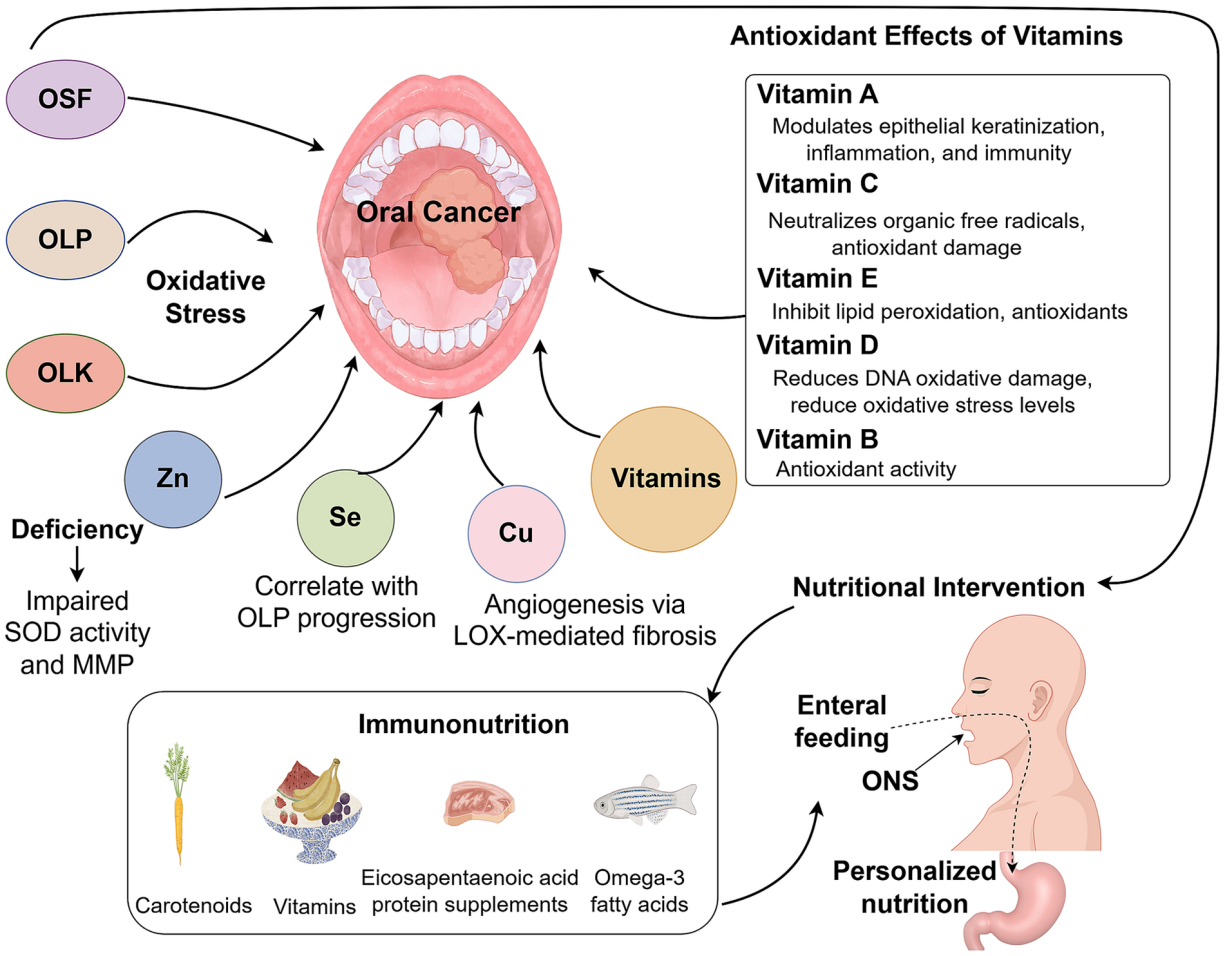
Role of micronutrition in patients with oral cancer.

For oral cancer patients with preserved swallowing function, modifying food texture and increasing nutrient density can meet nutritional needs (92). Oral nutritional supplements (ONS) have been shown to improve nutrient intake and quality of life, although they do not significantly affect mortality rates (93). When combined with dietary counseling, ONS enhances micronutrient intake and helps maintain body weight. In patients with impaired oral intake, enteral feeding via nasogastric or gastrostomy tubes significantly improves immune function, clinical recovery, nutritional status, and reduces hospital stay duration (94). Personalized nutrition assessments offer dynamic, tailored interventions, leading to greater improvements in serum albumin levels, handgrip strength, and lower rates of gastrointestinal complications compared to standard approaches (15). Immunonutrition, involving targeted supplementation with amino acids, fatty acids, nucleotides, and vitamins, modulates immune cell activity, particularly natural killer (NK) cell function, and improves clinical outcomes. Nutrients such as carotenoids, vitamin E, selenium, n-6/n-3 fatty acids, and eicosapentaenoic acid protein supplements have shown potential in preventing oral cancer (95, 96). Specifically, omega-3 fatty acids and arginine have been associated with enhanced progression-free survival, improved serum protein levels, and higher lymphocyte counts (97). Moreover, immune-modulating formulas have been reported to reduce postoperative inflammation in oral cancer patients (Figure 1) (98).

## 4 Comparative efficacy and limitations of micronutrient and Immunonutrition interventions

While multiple micronutrients and immunonutritional components have demonstrated potential in the management of oral cancer, their relative efficacy, safety, and mechanistic strengths require further critical evaluation (99). Zinc supplementation is associated with enhanced mucosal repair and oxidative stress reduction, particularly in OSF and OLP (100); however, prolonged high-dose use may disrupt copper metabolism, leading to hypocupremia-induced anemia and immunosuppression (101). Selenium, especially in its organic forms such as selenomethionine, exhibits potent anti-inflammatory and redox-stabilizing effects, with clinical evidence supporting its comparability to corticosteroids in the short term and superiority in long-term symptom control in OLP (50). Nevertheless, its narrow therapeutic index limits broader applicability due to risks of selenosis. Vitamin A is effective in reversing precancerous lesions like OLK but poses significant toxicity risks during long-term use, including mucosal dryness and hepatotoxicity (102). In contrast, vitamin E shows a more favorable safety profile and may synergize with lycopene in OSF management (103); however, antagonistic interactions with vitamin A have been reported, indicating the need for empirical testing of combined regimens. Vitamin C displays a bimodal redox activity, acting as an antioxidant at physiological levels and a pro-oxidant at pharmacological concentrations, raising interest in its therapeutic role in selectively inducing cancer cell death, although its clinical application requires definition of safe dosing windows (104, 105). Vitamin D, through the VDR signaling pathway, offers consistent antioxidant and immunomodulatory benefits in oral mucosal disorders, with minimal adverse effects, making it a promising adjunct in managing inflammatory and neoplastic oral lesions (89, 90). Among broader nutritional strategies, immunonutrition, incorporating omega-3 fatty acids, arginine, and nucleotides have shown superior outcomes in reducing inflammation, preserving lean mass, and improving treatment tolerance relative to standard nutritional support (106, 107). While combined immunonutrition regimens appear more effective than isolated micronutrient supplementation, variability in formulations and dosages complicates cross-study comparisons, highlighting the need for standardized, multicenter trials to validate their clinical utility (104, 108).

## 5 Conclusion

The critical role of micronutrients in oral carcinogenesis and therapy is underscored by their dual capacity to modulate oxidative stress and inflammatory pathways. Deficiencies in zinc and selenium disrupt redox homeostasis, impairing SOD and GPX activity, while copper excess promotes fibrosis and angiogenesis in OSF. Vitamins A, C, and E demonstrate chemopreventive potential but require precise dosing to avoid antagonistic or pro-oxidant effects. Immunonutrition strategies, particularly those incorporating omega-3 fatty acids and arginine, show promise in enhancing treatment tolerance and immune function. However, the therapeutic window for many micronutrients remains narrow, necessitating further research to optimize dosing regimens.

Future studies should focus on biomarker-guided supplementation to enable personalized nutrition interventions. Mechanistic investigations into vitamin D’s role in mucosal repair via VDR signaling may offer novel therapeutic avenues. Additionally, standardized protocols for combined antioxidant therapies are needed to maximize efficacy while minimizing adverse effects. Integrating these nutritional approaches with conventional treatments could improve clinical outcomes and quality of life for oral cancer patients, bridging a critical gap in comprehensive cancer care.
